# Gene Expression Analysis in Four Dogs With Canine Pemphigus Clinical Subtypes Reveals B Cell Signatures and Immune Activation Pathways Similar to Human Disease

**DOI:** 10.3389/fmed.2021.723982

**Published:** 2021-09-29

**Authors:** Haya S. Raef, Cesar Piedra-Mora, Neil B. Wong, Diana Junyue Ma, Clement N. David, Nicholas A. Robinson, Ramón M. Almela, Jillian M. Richmond

**Affiliations:** ^1^Department of Dermatology, UMass Medical School, Worcester, MA, United States; ^2^Tufts University School of Medicine, Boston, MA, United States; ^3^Clinical Sciences Department, Tufts Cummings School of Veterinary Medicine, Grafton, MA, United States; ^4^NanoString Technologies Inc., Seattle, WA, United States

**Keywords:** canine (dog), pemphigus, autoimmune blistering diseases, gene expression, NanoString, cytokine, skin, comparative immunology

## Abstract

Pemphigus is a group of autoimmune-mediated mucocutaneous blistering diseases characterized by acantholysis. Pemphigus has also been recognized in dogs and shares similar clinical characteristics and variants with human pemphigus. While relationships between human and canine pemphigus have been reported, gene expression patterns across species have not been described in the literature. We sought to perform gene expression analysis of lesional skin tissue from four dogs with various forms of pemphigus to examine gene expression during spontaneous disease in dogs. We found increased T and B cell signatures in canine pemphigus lesions compared to controls, as well as significant upregulation of *CCL3, CCL4, CXCL10*, and *CXCL8 (IL8)*, among other genes. Similar chemokine/cytokine expression patterns and immune infiltrates have been reported in humans, suggesting that these genes play a role in spontaneous disease. Direct comparison of our dataset to previously published human pemphigus datasets revealed five conserved differentially expressed genes: *CD19, WIF1, CXCL10, CD86*, and *S100A12*. Our data expands our understanding of pemphigus and facilitates identification of biomarkers for prediction of disease prognosis and treatment response, which may be useful for future veterinary and human clinical trials.

## Introduction

Pemphigus is a group of potentially life-threatening autoimmune blistering diseases characterized by acantholysis. In these diseases, IgG autoantibodies target intraepithelial adhesion molecules resulting in epidermal splitting and blister formation (1). The incidence of pemphigus varies substantially around the world and is estimated to range between 0.75 and 5 new cases per million (2, 3).

Pemphigus can be categorized into different subtypes including pemphigus vulgaris and pemphigus foliaceus, depending on the level of blistering in the epidermis. In pemphigus foliaceous, the disease is restricted to the upper part of the epidermis, whereas in pemphigus vulgaris, deeper layers of the epidermis are involved resulting in a more erosive presentation (4, 5). In the United States, pemphigus vulgaris is the most common subtype, primarily affecting women between 50 and 60 years of age (2, 6). Pemphigus vulgaris is thought to result from autoantibody action against desmosomal components, primarily desmoglein (Dsg) 1 and Dsg 3, leading to acantholysis (7).

Pemphigus has also been recognized in dogs and shares similar clinical characteristics and variants with human pemphigus (8). While pemphigus vulgaris is the most prevalent subtype of pemphigus in humans, it is uncommon in dogs (9). Pemphigus foliaceus is believed to be the most common variant in dogs (9). It is most often caused by autoantibodies targeting desmocollin-1 (10), which is in contrast to human pemphigus foliaceus disease, in which desmoglein-1 (DSG1) represents the major autoimmune target (1, 11). In canine pemphigus foliaceus, but less commonly in humans, transient pustules develop, which rapidly evolve into yellowish crusts and erosions due to neutrophilic infiltration that precedes acantholysis (8, 12). In dogs, lesions are often localized to the face or footpad, and later progress into generalized distribution. Pemphigus erythematosus is considered a variant of pemphigus foliaceus, with overlapping immunologic and clinical features of both lupus and pemphigus entities (13). Canine pemphigus erythematous lesions are characterized by acantholytic pustules along with a lichenoid interface dermatitis that is commonly restricted to the face.

While similarities between human and canine pemphigus have been reported, gene expression patterns across species have not been described in the literature. An advantage of studying spontaneous pemphigus in dogs is that it eliminates the layer of potential bias inherent in constructing an inducible animal model. We analyzed lesional skin tissue using NanoString technology from four dogs with various forms of pemphigus who presented to community veterinary clinics and were biopsied for diagnostic purposes. We present gene expression analyses from this case series, providing further evidence that canine pemphigus closely mimics the human condition. Our goal is to help identify novel target genes for further study as both biomarkers and drivers of pathogenesis.

## Materials and Methods

### Clinical Samples

The animal study was reviewed and approved by the Institutional Animal Care and Use Committee (IACUC) at the Tufts University Cummings School of Veterinary Medicine. Specimens from veterinary patients were collected when seen at the Cummings School of Veterinary Medicine, and stored in the biorepository after obtaining written consent from owners.

Skin biopsies demonstrating “pustular dermatitis” or “acantholytic dermatitis” between 2011 and 2019 were drawn from the biorepository, and potential cases were submitted to a veterinary dermatologist and pathologist for categorization into their appropriate diagnoses. Inclusion criteria for pemphigus samples included clinical presentation compatible with pemphigus disease, with morphological diagnoses of subcorneal, intragranular or subgranular acantholytic neutrophilic or mixed neutrophilic/eosinophilic pustules, and absence of proteolytic acantholytic dermatoses (staphylococcal and pustular dermatophyte infections of the skin) after histochemical stains (PAS and/or Gram). The different pemphigus subtypes were classified as follows:

- Pemphigus Foliaceous: Pustules rapidly evolving into shallow erosions and crusts with predominance to the face and feet. Histopathologic findings must include superficial epidermal, or follicular pustules rich in neutrophils with clustered acantholytic keratinocytes. Other acantholytic neutrophilic pustular diseases must be ruled out.
- Pemphigus Vulgaris: A clinical presentation that includes mucosal and/or cutaneous vesicles, bullae, deep erosions, or ulcers. Histopathology must show suprabasal epidermal, mucosal, or follicular acantholysis.
- Pemphigus Erythematosus: Pustules, erosions, and crusts localized to the face and pinnae, along with depigmentation, erythema, and erosion/ulceration of the nasal planum and dorsal muzzle. Histology morphological diagnosis should reveal intragranular to subcorneal neutrophilic and eosinophilic acantholytic pustules with a lichenoid interface dermatitis.

Immunologic tests were not performed on these animals; therefore, diagnoses were based principally on clinico-pathological grounds in accordance with Olivry et al. (8).

Control tissue were also obtained from the biorepository. These tissue samples were derived from canine healthy margin skin from leg amputations. The surgeons routinely excise an extra margin of tissue to look for malignancy, necrosis, etc. Histopathologic data from all cases were reexamined and validated by a board-certified veterinary pathologist and dermatologist (NAR, RMA). A summary of the clinical characteristics is included in the Case Presentations section.

### Isolation of RNA From Formalin-Fixed, Paraffin-Embedded (FFPE) Blocks

FFPE blocks were sectioned into 30 μm thick curls and stored in Eppendorf tubes at ambient temperature. RNA extraction and purification from FFPE blocks was conducted using the Qiagen FFPE RNeasy kit, according to manufacturer's instructions. Briefly, razor blades were treated with RNase, excess paraffin was trimmed, and tissues were sliced into thin 5 μm section before treatment with deparaffinization solution (Qiagen). RNA was assessed for quantity using a NanoDrop Spectrophotometer (NanoDrop Technologies, USA), which measures RNA concentration by calculating UV absorbance.

### Nanostring Cartridge, Processing, and Analysis

We quantified gene expression from canine pemphigus patients using a custom NanoString canine gene panel comprised of 160 genes involved in various immune pathways and skin homeostasis. We used *B2m, Rpl13a, cg14980*, and *hprt* as housekeeping genes. RNA hybridization was carried out using a BioRad C1000 touch machine. The hybridized samples were then loaded into NanoString cartridges and analyzed with a Sprint profiler. Data analysis was performed using nSolver software (14) and raw counts and DEGs were plotted using GraphPad Prism v9. Raw data are shared on Gene Expression Omnibus (GEO) Database under accession #GSE171079.

### H&E and IHC

Biopsy samples were formalin fixed and paraffin wax-embedded. Five-micron sections were either stained with haematoxylin and eosin (H&E) on an automated slide stainer (Sakura Tissue-Tek DRS), or stained for immunohistochemistry using rabbit anti-canine CXCL10 (US Biological, 1:100) using an automated staining machine (Dako EnVision + Dual Link System-HRP). Staining was visualized using a HRP conjugated secondary anti-rabbit antibody. Images were captured using an Olympus BX51 microscope equipped with Nikon NIS Elements software version 3.10.

### Canine and Human Dataset Comparison

To determine the number of conserved genes between human and canine pemphigus, we compared our dog NanoString dataset to a human pemphigus microarray dataset available on GEO database [GSE53873; (14)]. We first found the common denominator genes from both datasets using the “MATCH” function in Microsoft Excel, then truncated the datasets to reflect shared transcripts covered in each study (total 156 genes). We next examined the differentially expressed genes (DEGs) in each dataset with *p* < 0.05. To determine total overlap of significant DEGs shared by both human and canine pemphigus disease, we compared the DEG lists of both species and generated a Venn diagram using the BioVenn web application (15).

### Statistics

We performed statistical analysis to compare healthy to pemphigus lesional skin using nSolver software (NanoString, Seattle, WA) and GraphPad Prism software (version 9.0, GraphPad Software, San Diego, CA). Shapiro-Wilk tests were performed to check for normality. Data were analyzed with the two-tailed student's *t*-test if data were determined to be normally distributed, or with the two-tailed Mann Whitney *U*-test for non-normally distributed data. *P* < 0.05 were considered significant, and *P* < 0.01 were considered highly significant.

## Case Presentations

### Case 1

Ten-year-old spayed female Miniature Schnauzer that presented with a 3-week history of nasal discharge and a 10-day history of right front limb lameness. On physical examination, ulcerative lesions on the left nasal planum and right paw pad were observed, along with swelling of the tongue and palate. Biopsy of the hard palate, nasal planum and tongue demonstrated mucosal hyperplasia with suprabasilar clefting, focal ulceration and mild lymphoplasmacytic interface inflammation. Moreover, there was marked pigmentary incontinence in the nasal planum. These findings were most consistent with pemphigus vulgaris.

### Case 2

A 9-year-old spayed female Labrador Cross with a history of ulcerative stomatitis presented to the emergency room due to difficulty prehending food. On physical exam, ulcerations were noted along the face, ears, and inguinal skin. The area was very sensitive to touch. A skin biopsy was taken, revealing a thickened epidermis with acanthosis. In the stratum spinosum and stratum granulosum, there were ragged and ruptured foci and clear spaces, suggestive of vesicle formation. Along the dermo-epidermal junction, there was edema, pigmentary incontinence, and moderate inflammatory interface dermatitis. These findings were consistent with pemphigus erythematosus.

### Case 3

Seven-year-old female spayed German Shepherd Cross presented with a 1.5 year history of nasal and periocular depigmentation and crusting. Physical examination demonstrated thick yellow crusting on the ear flaps, around both eyes, above the nasal planum, and in multifocal areas along her flanks. She has previously responded well to steroids but has discontinued due to adverse side effects. Histopathologic evaluation showed moderate lymphoplasmacytic interface dermatitis with pigmentary incontinence. Moreover, marked eosinophilic and neutrophilic, subcorneal and exudative dermatitis with infrequent *ghost* acantholytic cells were observed. Clinical and histologic findings were consistent with pemphigus erythematosus.

### Case 4

Twelve-year-old male neutered Portugese Water dog presented with a 10 month history of nasal crusting, ulceration and allergic rhinitis. On physical exam, crusting was observed on the nasal planum with erosion and depigmentation. The canine patient was initially diagnosed with mucocutaneous pyoderma, however his condition was unresponsive to antibiotic treatments and later progressed to involve the foot pads. Histopathologic evaluation demonstrated suprabasilar neutrophilic pustules with rafts of acantholytic keratinocytes. Additionally, there was superficial dermal and interface inflammatory infiltrate composed of lymphocytes and plasma cells. These findings were consistent with pemphigus foliaceous disease.

## Results

### Key Immune Genes Are Upregulated in Canine Pemphigus vs. Healthy Controls

To begin to characterize gene expression in canine pemphigus, we isolated RNA from archival skin biopsies from four canine pemphigus patients, and five healthy controls. We used NanoString technology to analyze RNA from these samples as it quantifies genes without the needs of amplification and is useful for measuring the gene expression of difficult samples which cannot be easily amplified in PCR. It also has a considerable fast turnaround time and offers robust data.

**Figure 1:**
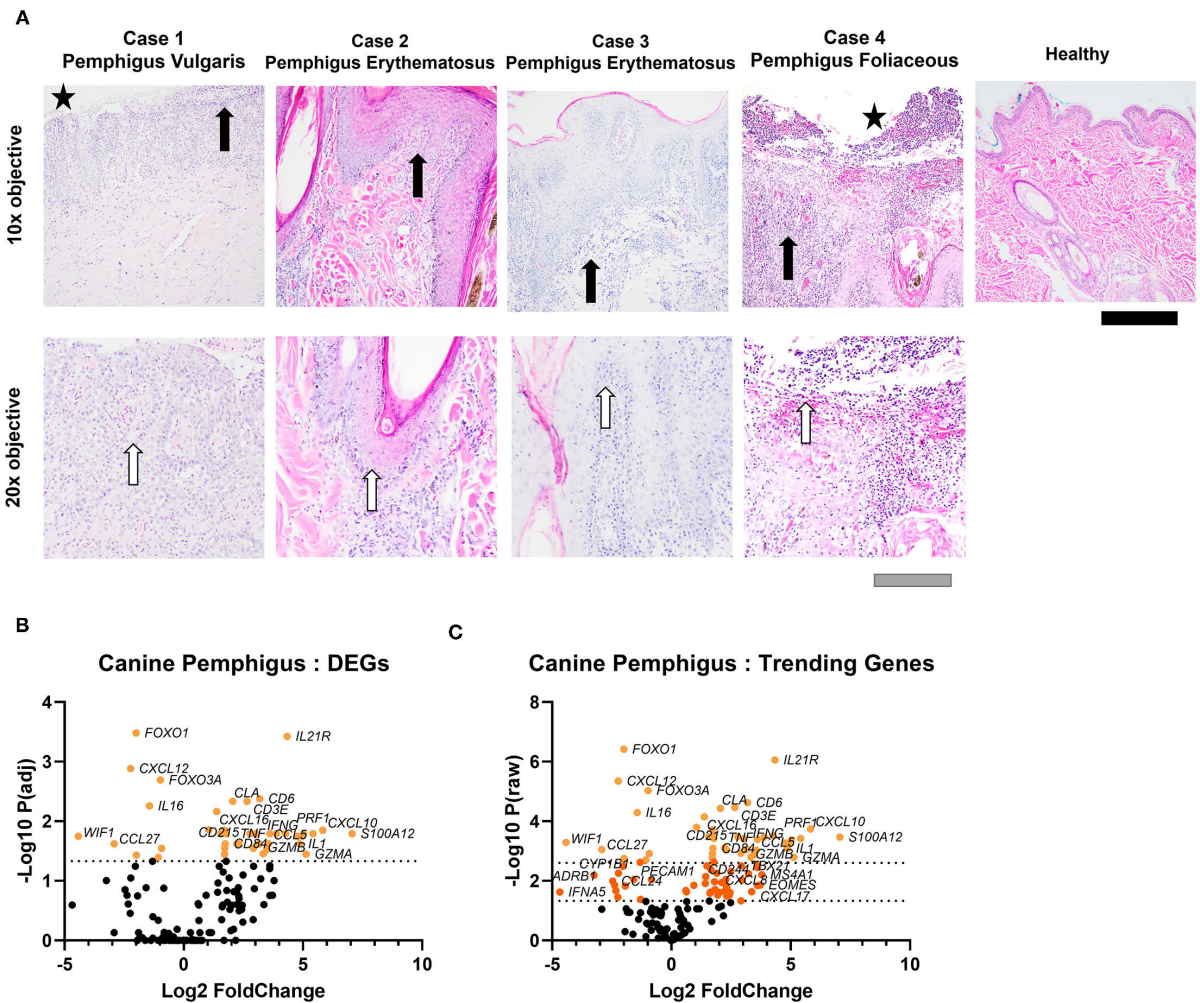
H&E staining of canine pemphigus and healthy canine skin reveals histopathologic features of pemphigus variants. **(A)** All cases revealed interface dermatitis, edema, and pigmentary incontinence at the dermo-epidermal junction (black arrows indicate interface dermatitis). Case 1, pemphigus vulgaris, and case 4, pemphigus foliaceous, revealed epidermal sloughing (black stars). Polymorphonuclear cells were noted in all biopsies (white arrows). Scale bars black = 100 μm, gray = 200 μm. **(B)** Volcano plot of differential gene expression (DEG) analysis in canine pemphigus vs. healthy skin margins using BY adjusted *P*-values or **(C)** raw *P*-values (*n* = 4 pemphigus cases and 5 healthy margin controls).

Utilizing the high-fidelity NanoString nCounter platform, we analyzed 160 sets of canine genes to compare gene expression patterns between pemphigus and healthy canine patients (H&E from cases presented in Figure 1A). Agglomerative clustering of the comprehensive set of genes discriminates between healthy and pemphigus canine skin (Supplementary Figure 1). Principal component analysis (PCA) showed that the transcriptomic profiles of pemphigus cases are distinguishable from healthy cases (Supplementary Figure 2). Out of the 160 genes, 43 genes were differentially expressed, using a Benjamini-Yekutieli (BY) adjusted *P* < 0.05 (Figure 1B). Nine of these genes had a negative log2 fold change (downregulated compared to healthy), and the remaining 34 genes had a positive log2 fold change (upregulated compared to healthy). We also examined genes that were trending toward significance, as these might provide insights into additional genes driving pathogenesis of canine pemphigus, but were under-powered in our study of four cases (Figure 1C). Ninety-four genes had a raw *P* < 0.05. Forty-six of these genes were upregulated with a log fold change of >2. Sixteen genes were downregulated with a log fold change < -1.5.

**Figure 2:**
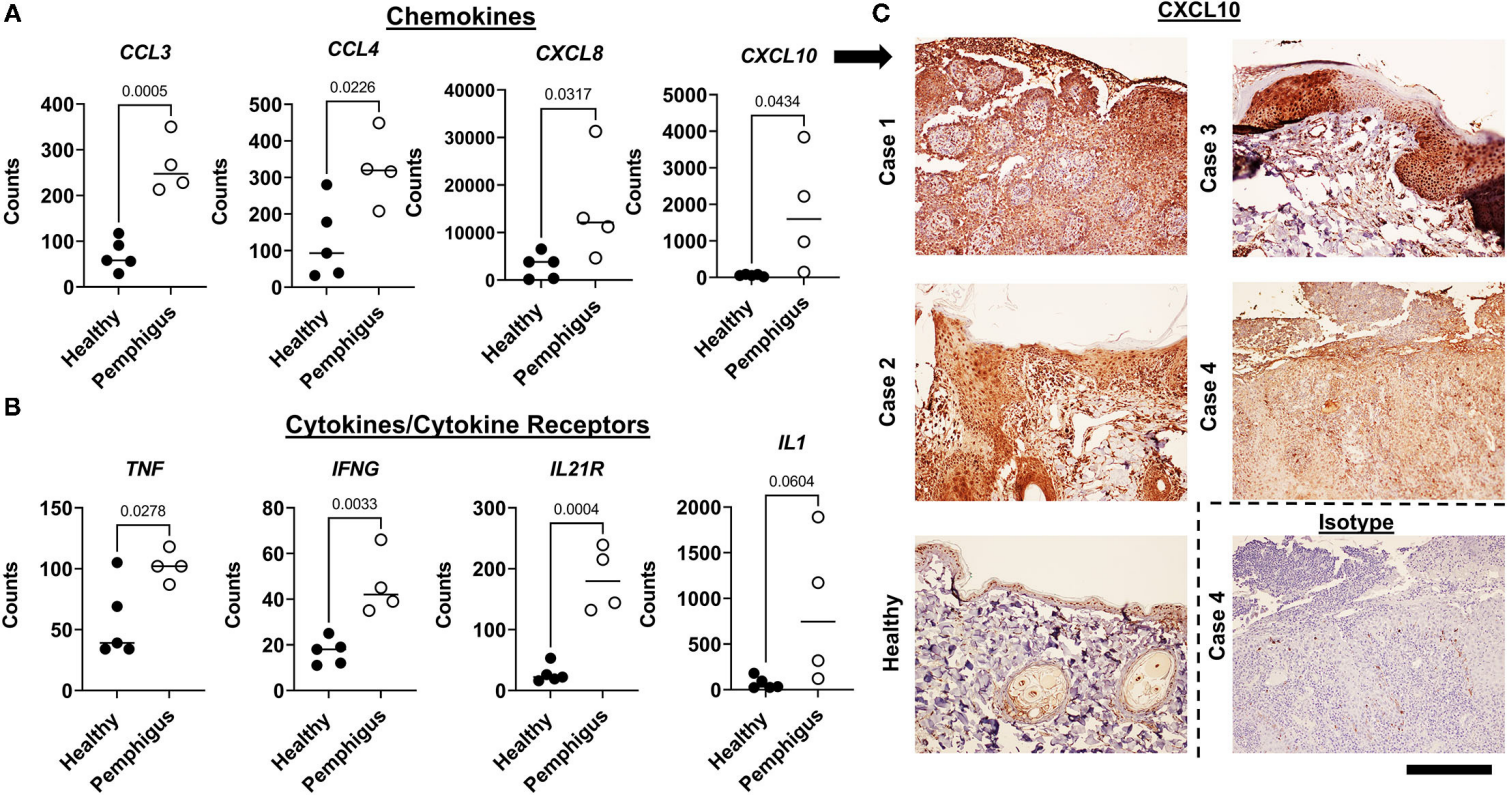
Examination of key inflammatory gene expression in canine pemphigus variants reveals conserved patterns similar to previously published human pemphigus datasets. **(A)** Gene expression of chemokines and **(B)** cytokines/receptors in canine skin biopsies that were previously identified to be upregulated in human pemphigus [Mann-Whitney *U*-tests (non-normally distributed data) and student's two tailed *t*-tests (normally distributed data) significant as indicated; *n* = 4 pemphigus and 5 healthy margins]. **(C)** Confirmation of CXCL10 expression with IHC in cases vs. healthy and isotype controls. 100x, Scale bar = 300 μm.

To understand the immunopathogenesis of canine pemphigus, we performed more detailed analysis on subsets of genes based on known cellular functions and previously published gene expression analyses of human pemphigus. First, we examined cytokines and chemokines. We found significant upregulation of the chemokines *CCL3, CCL4, CXCL10*, and *CXCL8 (IL8)* (Figure 2A). There was also significantly increased *TNF, IFNG*, and *IL21R* (though not *IL21* cytokine) expression compared to healthy controls (Figure 2B). Many of these cytokines or cytokine families have been previously reported to be induced in blistering disorders (16). There was a trend toward increased *IL1* expression, but this was not statistically significant by two tailed student's *t*-test (Figure 2B).

### CXCL10 Is Highly Expressed at the Protein Level in Canine Pemphigus Lesions

To confirm translation of key genes identified in our NanoString codeset, IHC was performed for CXCL10. Results showed that lesional skin biopsies from dogs demonstrated increased expression of CXCL10 compared to isotype and healthy controls (Figure 2C). CXCL10 was expressed throughout the epidermis and has been implicated in the pathogenesis of human pemphigus.

### Skin Homeostatic Genes Are Downregulated in Canine Pemphigus

We examined genes related to skin homeostasis, as well as immune regulatory and neuroendocrine genes in our canine dataset to understand more about the skin tissue microenvironment. Analysis of neuroendocrine genes revealed that *ADRB1, MCR5*, and *CYP1B1* were significantly downregulated in pemphigus vs. healthy controls (Supplementary Figure 3). *IGFBP5*, which is known to mediate fibrosis, and *FOXO1*, which is associated with adipogenesis, were significantly downregulated in lesional skin (Supplementary Figure 3). Several other skin homeostatic genes, including *WIF1, EDA, RXRG*, and *TGFB2*, were significantly decreased in pemphigus cases. The keratinocyte differentiation marker, involucrin *IVL*, was significantly enriched in pemphigus lesions. *KRT14*, a marker of keratinocytes, was significantly higher, and *PECAM1*, a marker of endothelial cells, was significantly lower in pemphigus cases vs. controls (Supplementary Figure 3).

### Cell Type Analysis Reveals Infiltration of Activated Lymphocytes and Granulocytes in Canine Pemphigus Lesions

**Figure 3:**
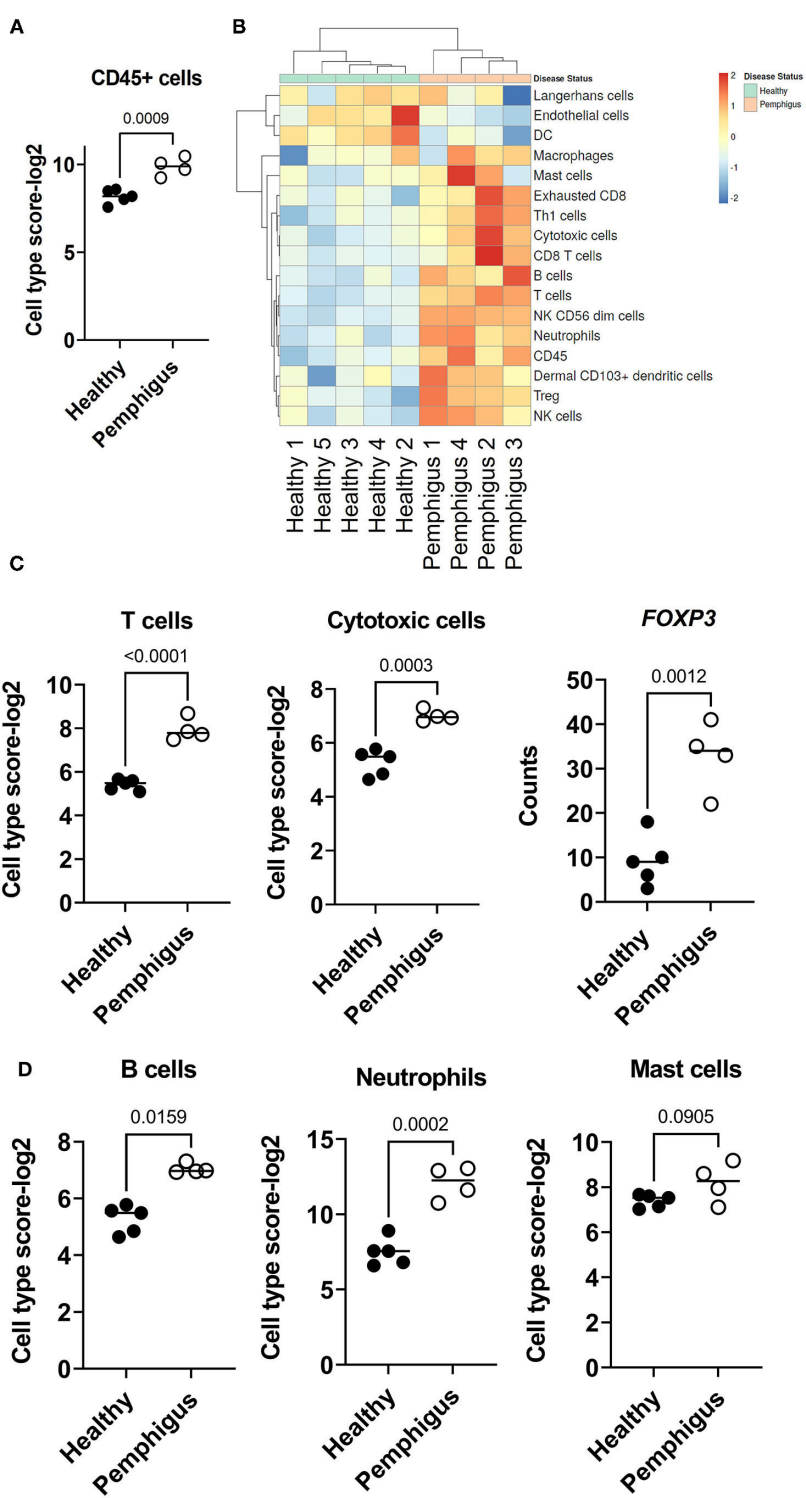
Cell type enrichment scores in canine pemphigus reveals infiltrations of T, B, NK cells, and neutrophils. **(A)** Total CD45+ infiltrates were significantly higher in cases vs. healthy margins. **(B)** Heatmap of cell type scores in healthy (green) and pemphigus (orange) samples. **(C)** Cell type scores from NanoString advanced analysis with high confidence of prediction (*P* < 0.01) demonstrating significant increases in T cells (*CD3E, SH2D1A, CD6, TRAT1* composite score), cytotoxic cells (*GZMA, KLRK1, PRF1, GZMB, KLRB1, KLRD1, CTSW* composite score), and Tregs (*FOXP3*) in canine pemphigus skin compared to healthy controls. **(D)** B cell (*CD19, MS4A1, BLK, FCRL2, TNFRSF1* composite score), neutrophil (*CEACAM1, S100A12, CSF3R* composite score) and Mast cell (*HDC, MS4A2, CPA3* composite score) cell type analysis scores had *P* < 0.05, indicating moderate confidence for prediction. B cells and neutrophils were significantly increased in canine pemphigus compared to healthy controls (*n* = 4 pemphigus cases and 5 healthy controls, two tailed student's *t*-tests significant as indicated).

There was a significant increase of *CD45*+ immune cells in the skin of canine pemphigus patients compared to controls (Figure 3A). To begin to characterize this inflammatory infiltrate, we used NanoString Advanced Analysis, a method that has been previously validated with flow cytometry, to determine which cellular gene signatures were present in canine pemphigus (17) (Figure 3B). Cell type enrichment analysis revealed that T cells, including cytotoxic T cells are highly significantly increased in canine pemphigus compared to healthy controls, with a log2 cell type enrichment score of *P* < 0.01 (Figure 3C). We examined expression of the transcription factor *FOXP3*, which serves as important master regulator of regulatory T cell (Treg) differentiation and found that it was upregulated in pemphigus compared to controls (Figure 3C). Mast cells, B cells, and Neutrophils each had a log2 cell type enrichment score of *P* = 0.05, which means their presence in tissue has a moderate accuracy for prediction. B cells and neutrophils were increased in canine pemphigus skin (Figure 3D).

### Comparative Analysis of Canine and Human Pemphigus Reveals Shared Differentially Expressed Genes

**Figure 4:**
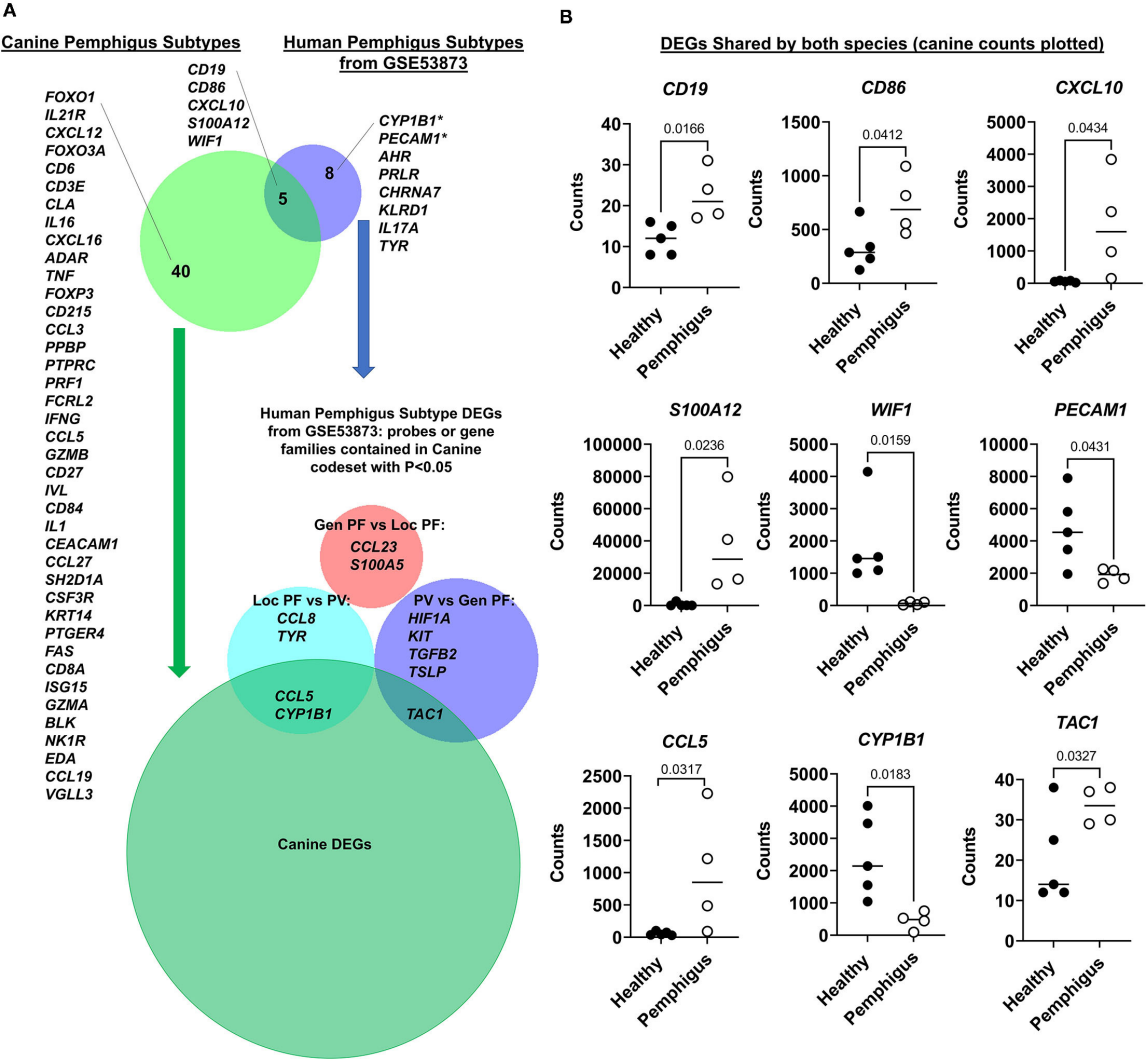
Comparative analysis of human pemphigus cases from GSE53873 and canine pemphigus cases reveals shared DEGs. **(A)** Analysis of DEGs from pooled, untreated human pemphigus subtypes from GSE53873 and canine pemphigus subtypes. Further analysis of DEGs between human pemphigus subtypes as determined using GEO2R Venn Diagram function revealed that canine pemphigus captures elements of both human localized pemphigus foliaceous and pemphigus vulgaris, but not generalized pemphigus foliaceous. **(B)** Canine RNA counts for DEGs shared by both species are presented, including *CYP1B1* and *PECAM1*, which were trending toward significance in the overall canine differential expression analysis. *CYP1B1 and *PECAM1* are significant when directly compared between canine pemphigus and healthy margins using two-tailed student's *t*-tests (Gen PF, generalized pemphigus foliaceous; Loc PF, localized pemphigus foliaceous; PV, pemphigus vulgaris; DEGs, differentially expressed genes).

We next examined shared gene expression patterns between human and canine pemphigus. We selected all untreated samples from dataset GSE53873 (16), which included skin biopsies from human pemphigus vulgaris and foliaceous patients with both localized and generalized disease, as our four canine cases also exhibited clinical heterogeneity. We first truncated both datasets to a list of common denominator gene transcripts that were captured in both platforms, which yielded a list of 156 genes. Next, we examined which of these common denominator genes were differentially expressed in each species, and then used BioVenn software to compare the significant DEGs for human and canine. Our analysis demonstrated five DEGs that are conserved in all clinical subtypes of human and canine pemphigus disease: *CD19, WIF1, CXCL10, CD86*, and *S100A12*. *CYP1B1* and *PECAM1*, which were trending toward significance in our canine dataset using raw *P* < 0.05 from differential expression analysis, were significant DEGs in the human dataset. Differentially expressed genes in humans and canine pemphigus are summarized in Figure 4A.

To determine if any clinical subtypes share common genes, we next used the human pemphigus subtype DEGs from GSE53873 generated using the GEO2R Venn Diagram function, and we determined which, if any, DEGs were contained in the canine codeset with *P* < 0.05. We found that genes enriched in localized human pemphigus foliaceous compared to pemphigus vulgaris, including *CCL5* and *CYP1B1* were also captured in the canine DEGs when examined by two-tailed student's *t*-tests (Figure 4B). *TAC1* (substance *P* gene) was enriched in human pemphigus vulgaris compared to generalized pemphigus foliaceous, and this was also a DEG in our canine dataset. Generalized human pemphigus appears to be the least similar to canine pemphigus variants, though this may be due to our limited sample size or the clinical subtypes we examined.

## Discussion

The goals of our study were to examine immune and skin gene expression in canine pemphigus to (1) better map immunopathogenesis in veterinary patients and (2) compare to human pemphigus to understand conserved drivers of disease. Our data showed that 94 out of the 160 quantified genes demonstrated statistically significant altered mRNA expression between pemphigus and healthy canine skin. The functions of these genes vary but include both immunological and skin barrier function. We found increased expression of genes encoding T and B cell associated cytokines, chemokines, neutrophil attractants, and immune mediators.

Pemphigus is caused by autoantibodies directed against self-proteins in the skin. These autoantibodies are thought to be generated by autoreactive B cells following T cell help. As in human pemphigus, we observed both CD3+ T cells and CD19+ B cells are increased in canine pemphigus lesions (18, 19). It has been suggested that pemphigus is a T helper (Th) type 2-dependent disorder (20, 21). Other T-cell subsets, including CD4+CD25+ regulatory T cells and Th17 cells, have also been implicated in the pathogenesis of pemphigus (22, 23). *FOXO1*, a known immunoregulatory gene, was decreased in our canine cases compared to healthy controls, which may indicate reduced ability of Tregs to function to suppress autoreactive T cells in pemphigus lesions. T follicular helper cells (Tfh) have been recently reported to be critically involved in the pathogenesis of pemphigus by facilitating B-cell activation and promoting autoantibody production (24, 25). Further studies are needed to confirm if similar T-cell subset patterns exist in canine pemphigus patients.

Additionally, we noted a trend toward increased mast cells in canine pemphigus skin lesions. Increased numbers of mast cells have been detected in a prior study in the skin of pemphigus vulgaris and bullous pemphigoid patients (26–28). While the role of mast cells in the pathogenesis of pemphigus is not entirely clear, it has been suggested that mast cells may contribute to acantholysis by inducing release of esterases. Moreover, high concentrations of Dsg3-reactive IgE and intercellular IgE deposits in pemphigus vulgaris patients have been reported, further supporting the involvement of mast cells in pemphigus (29).

Recent studies have suggested a critical role for IL-17 in the pathogenesis of human pemphigus disease (19, 24). IL-17 is produced by Th17 cells and is commonly associated with the development of autoimmune conditions (30–32). While we noted increased mRNA expression of *IL17* in only one canine pemphigus case (case 4), we did note increased *IL21R*. IL-21 cytokine is directly associated with the Th17 pathway, and prior studies have demonstrated increased levels of IL-21 in human pemphigus disease. IL-21 is a cytokine secreted mainly by Tfh and Th17 cells and has been suggested to stimulate B-cell activation and differentiation, leading to pathogenic autoantibody production in pemphigus lesions (19, 33). It is possible that the signal of *IL17* and/or *IL21* from T cells is diluted by analyzing bulk mRNA from skin tissue. Therefore, future studies immunophenotyping canine pemphigus T cells by flow cytometry, single cell RNA sequencing, spatial transcriptomics or similar enrichment approaches is warranted.

We also found significant upregulation of chemokines *CXCL8* and *CXCL10* compared with the normal skin controls. *CXCL8* and *CXCL10* mediate recruitment of leukocytes to the skin from the blood (34, 35), and have been implicated in the pathogenesis of human pemphigus (36, 37). CXCL10 has been shown to be highly expressed in human bullous pemphigoid disease, and is suggested to induce matrix metalloproteinase 9 (MMP), which is a key proteinase in the pathologic process associated with blister formation (36). Similarly, *CXCL8* has been hypothesized to recruit neutrophils to the epidermis and contribute to the blistering process (38).

Consistent with human studies (37, 39), *TNF* was also upregulated in pemphigus lesional skin. TNF functions to regulate acute phase proteins and induces secretion of several cytokines and chemokines (37). It also stimulates T-cells, modulates differentiation of B cells, and promotes endothelial cells to secrete neutrophil chemotactic factor to increase neutrophil migration into tissues (40–42). Thus, TNF may play a mediator role in pemphigus lesions by increasing epithelial damage.

We also examined skin-specific genes that are associated with skin biology. We found that lesional skin from dogs with pemphigus exhibited downregulation of genes involved in skin homeostasis and immune tolerance. We observed decreased *WIF-1* expression in pemphigus skin which was conserved across both canine and human datasets. The Wnt proteins have been shown to have a fundamental role in tissue homeostasis, including development of hair follicles and initiation of the anagen phase (43). Impaired Wnt signaling has been reported in psoriatic skin disease (44).

Limitations in our study include the small sample size, and number of genes analyzed (160 genes). Future studies should include larger scale comparative analyses and whole transcriptome sequencing to allow for a better understanding of the pathogenesis of pemphigus across species. Despite these limitations, we observed many differentially expressed genes that match previously published literature regarding potential drivers of pemphigus immunopathogenesis. To our knowledge, this is the first study that provides gene expression analysis for canine pemphigus immune and skin transcripts. We have shown that dogs capture features of human disease, which may benefit patient care by providing additional predictive models for human disease mechanisms. In light of the fact that CAR-T clinical veterinary and human trials are in development, we hope that our study will add potential biomarkers that could mutually benefit both species.

## Data Availability Statement

The datasets presented in this study can be found in online repositories. The names of the repository/repositories and accession number(s) can be found below: https://www.ncbi.nlm.nih.gov/geo/, GSE171079.

## Ethics Statement

The animal study was reviewed and approved by Tufts Cummings School of Veterinary Medicine IACUC. Written informed consent was obtained from the owners for the participation of their animals in this study.

## Author Contributions

JR: conceptualization, project administration, and funding acquisition. NR and JR: methodology and resources. CD: software. RA and CP-M: validation. HR, NW, and JR: formal analysis. RA, CP-M, NR, DM, JR, and CD: investigation. NR, CP-M, RA, and JR: data curation. HR: writing–original draft. HR, CP-M, NW, and JR: visualization. JR, NR, and RA: supervision. All authors writing–review and editing.

## Funding

JR was supported by a Career Development Award from the Dermatology Foundation.

## Conflict of Interest

JR is an inventor on use patents for targeting CXCR3 (0#15/851, 651) and IL15 (# 62489191) for the treatment of vitiligo. NR is an employee of bluebird bio. CD is an employee of NanoString. The remaining authors declare that the research was conducted in the absence of any commercial or financial relationships that could be construed as a potential conflict of interest.

## Publisher's Note

All claims expressed in this article are solely those of the authors and do not necessarily represent those of their affiliated organizations, or those of the publisher, the editors and the reviewers. Any product that may be evaluated in this article, or claim that may be made by its manufacturer, is not guaranteed or endorsed by the publisher.

## Supplementary Material

Supplementary Figure 1 Heatmap of all samples. Rows are centered; unit variance scaling is applied to rows. Both rows and columns are clustered using correlation distance and average linkage. 160 rows, 8 columns.

Supplementary Figure 2 PCA plot of samples. Unit variance scaling is applied to rows; SVD with imputation is used to calculate principal components. X and Y axis show principal component 1 and principal component 2 that explain 36.8 and 29.3% of the total variance, respectively. Prediction ellipses are such that with probability 0.95, a new observation from the same group will fall inside the ellipse. *N* = 8 data points.

Supplementary Figure 3 Skin and immune homeostatic genes are decreased in canine pemphigus compared to healthy control skin. Gene expression of skin and immune homeostatic genes in cases compared to controls (Mann-Whitney *U*-tests (non-normally distributed data) or two tailed student's *t*-tests (normally distributed data) significant as indicated; *n* = 4 pemphigus and 5 healthy margins).
