# Supplementary figures and images for: Gene Expression Analysis in Four Dogs With Canine Pemphigus Clinical Subtypes Reveals B Cell Signatures and Immune Activation Pathways Similar to Human Disease
Source: Front Med (Lausanne). 2021 Sep 29;8:723982. doi: 10.3389/fmed.2021.723982 (PMC8511432; doi:10.3389/fmed.2021.723982)

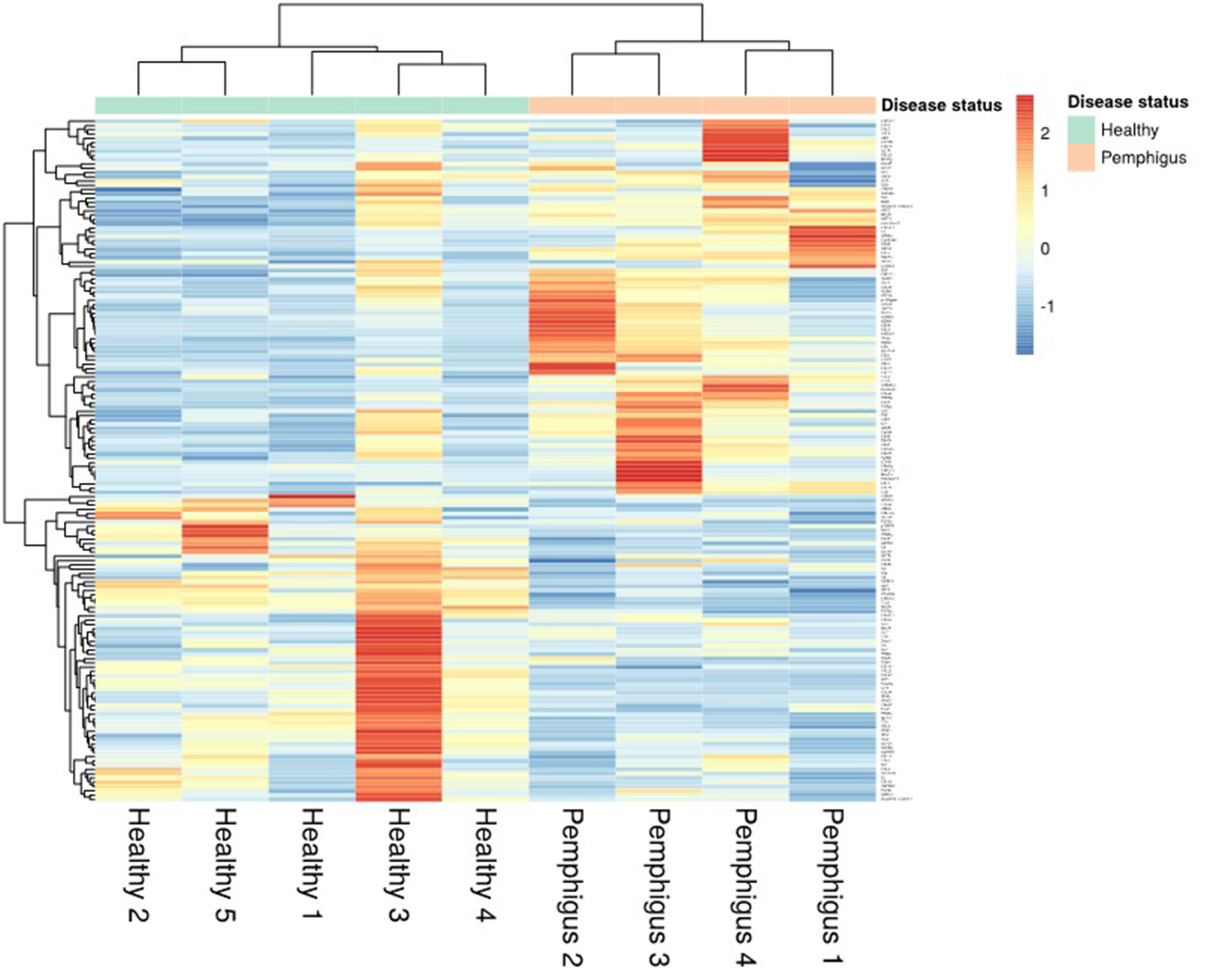

Supplement: Supplementary Figure 1 — Heatmap of all samples. Rows are centered; unit variance scaling is applied to rows. Both rows and columns are clustered using correlation distance and average linkage. 160 rows, 8 columns. [file Image_1.JPEG]

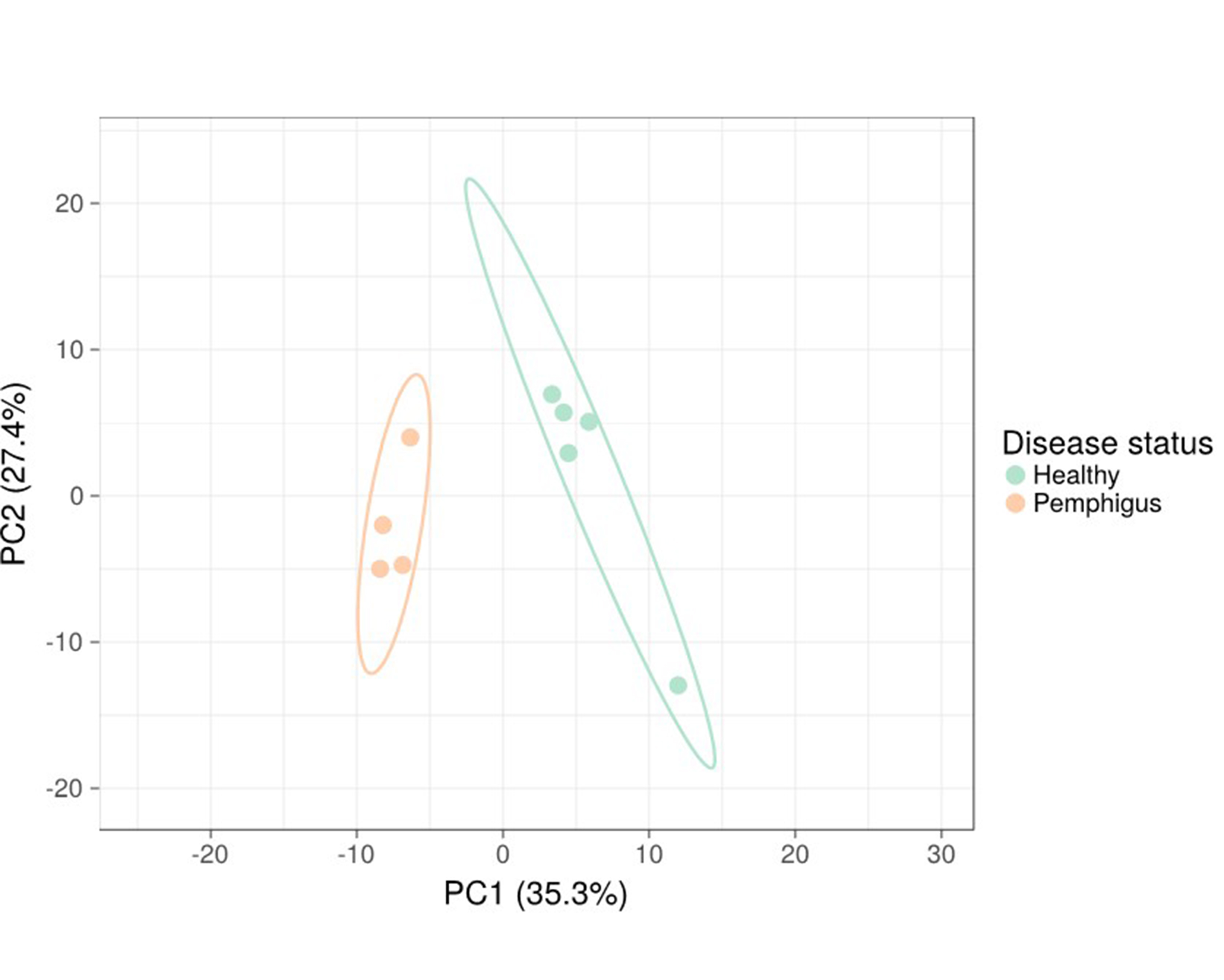

Supplement: Supplementary Figure 2 — PCA plot of samples. Unit variance scaling is applied to rows; SVD with imputation is used to calculate principal components. X and Y axis show principal component 1 and principal component 2 that explain 36.8 and 29.3% of the total variance, respectively. Prediction ellipses are such that with probability 0.95, a new observation from the same group will fall inside the ellipse. N = 8 data points. [file Image_2.JPEG]

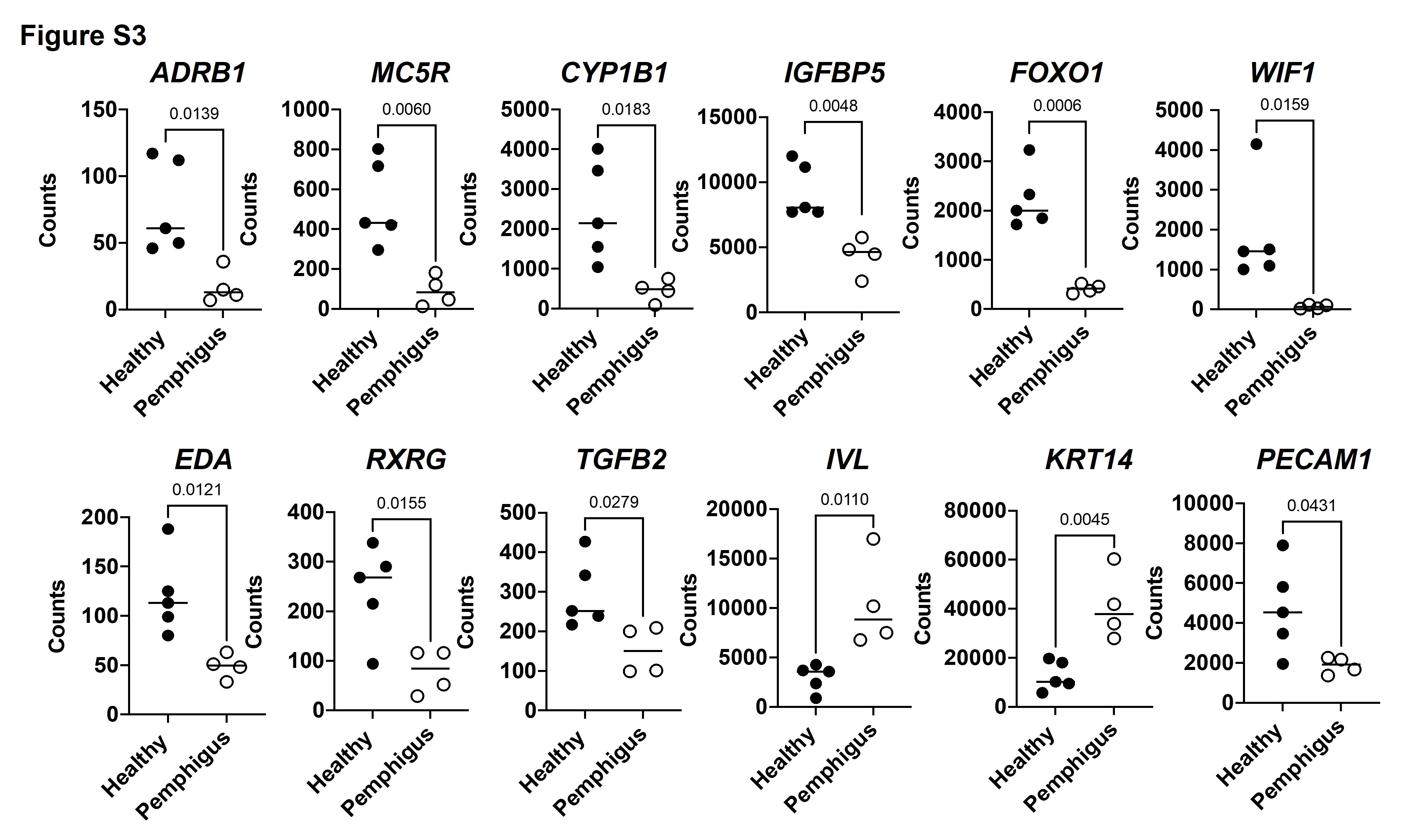

Supplement: Supplementary Figure 3 — Skin and immune homeostatic genes are decreased in canine pemphigus compared to healthy control skin. Gene expression of skin and immune homeostatic genes in cases compared to controls (Mann-Whitney U-tests (non-normally distributed data) or two tailed student's t-tests (normally distributed data) significant as indicated; n = 4 pemphigus and 5 healthy margins). [file Image_3.jpg]
